# Neutralizing antibodies against SARS-CoV-2 are higher but decline faster in mRNA vaccinees compared to individuals with natural infection

**DOI:** 10.1093/jtm/taac130

**Published:** 2022-11-07

**Authors:** Haissam Abou-Saleh, Bushra Y Abo-Halawa, Salma Younes, Nadin Younes, Duaa W Al-Sadeq, Farah M Shurrab, Na Liu, Hamda Qotba, Nader Al-Dewik, Ahmed Ismail, Hadi M Yassine, Laith J Abu-Raddad, Gheyath K Nasrallah

**Affiliations:** Biological Science Program, Department of Biological and Environmental Sciences, College of Arts and Science, Qatar University, Doha, Qatar; Biomedical Research Center, Qatar University, Doha, Qatar; Biological Science Program, Department of Biological and Environmental Sciences, College of Arts and Science, Qatar University, Doha, Qatar; Biomedical Research Center, Qatar University, Doha, Qatar; Biomedical Research Center, Qatar University, Doha, Qatar; Biomedical Research Center, Qatar University, Doha, Qatar; College of Medicine, Q.U. Health, Qatar University, Doha, Qatar; Biomedical Research Center, Qatar University, Doha, Qatar; Shenzhen Mindray Bio-Medical Electronics Co., Ltd, Shenzhen, China; Department of Clinical Research, Primary Health Care Centers, Doha, Qatar; Department of Pediatrics, Clinical and Metabolic Genetics, Hamad Medical Corporation, Doha, Qatar; Laboratory Section, Medical Commission Department, Ministry of Public Health, Doha, Qatar; Biomedical Research Center, Qatar University, Doha, Qatar; Department of Biomedical Science, College of Health Sciences, QU Health, Qatar University, Doha, Qatar; Infectious Disease Epidemiology Group, Weill Cornell Medicine – Qatar, Cornell University, Qatar Foundation – Education City, Doha, Qatar; World Health Organization Collaborating Centre for Disease Epidemiology Analytics on HIV/AIDS, Sexually Transmitted Infections, and Viral Hepatitis, Weill Cornell Medicine – Qatar, Cornell University, Qatar Foundation – Education City, Doha, Qatar; Department of Healthcare Policy and Research, Weill Cornell Medicine, Cornell University, New York, USA; Biomedical Research Center, Qatar University, Doha, Qatar; Department of Biomedical Science, College of Health Sciences, QU Health, Qatar University, Doha, Qatar

**Keywords:** mRNA vaccines, waning, neutralizing antibody, Anti-S1-IgA, SRBD-IgM

## Abstract

**Background:**

Waning protection against emerging SARS-CoV-2 variants by pre-existing antibodies elicited because of current vaccination or natural infection is a global concern. Whether this is due to the waning of immunity to SARS-COV-2 remains unclear.

**Aim:**

We aimed to investigate the dynamics of antibody isotype responses amongst vaccinated naïve (VN) and naturally infected (NI) individuals.

**Methods:**

We followed up antibody levels in COVID-19 messenger RNA (mRNA)-vaccinated subjects without prior infection (VN, *n* = 100) in two phases: phase-I (P-I) at ~ 1.4 and phase-II (P-II) at ~ 5.3 months. Antibody levels were compared with those of unvaccinated and naturally infected subjects (NI, *n* = 40) at ~ 1.7 (P-1) and 5.2 (P-II) months post-infection. Neutralizing antibodies (NTAb), anti-S-RBD-IgG, -IgM and anti-S-IgA isotypes were measured.

**Results:**

The VN group elicited significantly greater antibody responses (*P* < 0.001) than the NI group at P-I, except for IgM. In the VN group, a significant waning in antibody response was observed in all isotypes. There was about an ~ 4-fold decline in NTAb levels (*P* < 0.001), anti-S-RBD-IgG (~5-fold, *P* < 0.001), anti-S-RBD-IgM (~6-fold, *P* < 0.001) and anti-S1-IgA (2-fold, *P* < 0.001). In the NI group, a significant but less steady decline was notable in S-RBD-IgM (~2-fold, *P* < 0.001), and a much smaller but significant difference in NTAb (<2-fold, *P* < 0.001) anti-S-RBD IgG (<2-fold, *P* = 0.005). Unlike the VN group, the NI group mounted a lasting anti-S1-IgA response with no significant decline. Anti-S1-IgA, which were ~ 3-fold higher in VN subjects compared with NI in P-1 (*P* < 0.001), dropped to almost the same levels, with no significant difference observed between the two groups in P-II.

**Conclusion:**

Whereas double-dose mRNA vaccination boosted antibody levels, vaccinated individuals’ ‘boost’ was relatively short-lived.

## Introduction

The recent Coronavirus disease 19 (COVID-19) outbreak caused by the Severe Acute Respiratory Syndrome Coronavirus-2 (SARS-CoV-2) has put the globe in an emergency state.^1^ Messenger RNA (mRNA) vaccines, including Pfizer BNT162b2 and Moderna mRNA-1273, are amongst the approved vaccines by WHO and the US Federal Food and Drug Administration (FDA).^2^

On 21 December 2020, Qatar initiated a mass COVID-19 vaccination programme, first utilizing the BNT162b2 vaccine and then the mRNA-1273 (Moderna) vaccine.^3–7^ Both vaccinations were administered according to the FDA protocol, and vaccine coverage increased progressively from December 2020 until the time of writing.^3^^,^^5^ Vaccination was rolled out in stages, with frontline health care providers, those with severe or multiple chronic diseases, and people over 70 considered high priority. As of 26 February 2022, it was reported that at least 88% of people aged 12 and over were fully vaccinated.^8^

When vaccination was ramped up, the country had two back-to-back waves from January to June 2021, mainly dominated by the B.1.1.7 (or alpha) and B.1.351 (or beta) variants.^4^^,^^6^^,^^9–12^ The B.1.617.2 (or delta) variation was first spotted in Qatar around the end of March 2021, and by the summer of 2021, it had become the prevalent variety.^10–12^

According to current evidence, patients vaccinated against COVID-19 would lose around half of their neutralizing antibodies (NTAb) ~ 108 days post-vaccination.^13^ Moreover, recent research suggests that natural infections protect against re-infection for at least 8–12 months and that vaccination provides substantial protection from the Delta variant.^14^ However, despite widespread vaccination, the number of people infected with SARS-CoV-2 increased gradually in Qatar, with a significant 10-fold increase in the number of new COVID-19 cases in just two weeks, starting 29 December 2021, till 12 January 2022, during which Qatar hit an all-time high number of new daily cases.^15^ It is noteworthy that the Omicron (B.1.1.529) wave in Qatar started on 19 December 2021. Within 4 weeks, Omicron became the predominant strain, which could have contributed to the high numbers of re-infections.^16–18^ Therefore, protection against the new variants with pre-existing antibodies from natural infection or vaccination has become a huge concern.

Although vaccine-induced immunity is being extensively studied, the body of evidence for infection-induced immunity is very limited and insufficient to establish an antibody titre threshold that shows whether a person is protected from infection.^19^ In addition, there is currently no FDA-approved serology test that clinicians or the general public may perform to establish if a person is protected from infection.^19^ Therefore, this study aimed to investigate the dynamics of antibody response amongst mRNA-vaccinated naïve (VN) and naturally infected (NI) individuals in Qatar.

## Materials and Methods

### Ethical approval and sample collection

The study was reviewed and approved by the Institutional Review Board at Qatar University (QU-IRB 1537-FBA/21). The study included VN participants (*n* = 100) who had received two doses of the two approved mRNA vaccines in Qatar: BNT162b2 and mRNA-1273. Levels were compared with those of unvaccinated but NI subjects (*n* = 40). Venous blood samples were collected from vaccines in two phases: phase 1 (P-I) and phase (P-II). For the VN group, P-I samples were collected at 1.4 months (median = 6 weeks) and P-II samples were collected at 5.3 months (median = 23 weeks) after the second dose. For the NI group, P-I samples were collected at ~ 1.7 months (median = 7 weeks) and P-II samples were collected at 5.2 months (median = 22 weeks) post-infection with SARS-CoV-2.

Samples were centrifuged, and the plasma was separated from the whole blood and stored at −80^o^ C until performing the tests. Participants signed informed consent before sample collection, and each participant filled out a questionnaire about their demographic information in addition to questions related to any previous illness, including COVID-19 infection.

### Serology testing

After blood collection, plasma was separated by centrifugation and heat-inactivated. Serological testings were performed using the automated analyser CL-900i® from Mindray Biomedical Electronics 20-22 to detect: (1) NTAb (SARS-CoV-2 Neutralizing Antibody 121, Mindray, China) were measured using the automated analyser CL-900i®. This assay is a competitive binding chemiluminescent immunoassay for the quantitative determination of SARS-CoV-2 NTAb that blocks the interaction between the receptor binding domain (RBD) of viral spike protein (bound on magnetic beads) and the enzyme-conjugated ACE2 surface receptor. Phosphate-buffered saline (PBS) was used to dilute the samples with readings higher than the mentioned range. The assay has a WHO conversion factor of 1 AU = 3.31 IU/mL, and the reference range is 10 AU/mL to 400 AU/ml. We recently evaluated this novel assay and reported high specificity and sensitivity against two reference methods.^20^ (2) Antibodies to the RBD of the S1 subunit of the viral spike protein (IgG (S-RBD)) (Cat. No. SARS-CoV-2 Anti-S-RBD IgG122, Mindray, China), with a cut-off index for the kit is ≥10–1000 AU/ml, and WHO standardization factor of 1.15 BAU/mL. (3) IgA against a recombinant S1 domain of the SARS-CoV-2 was detected using Euroimmun Anti-SARS-CoV-2-ELISA (IgA) (EUR S-IgA) (Cat. No. EI 2606–9601A). The IgA ratio was calculated by dividing the extinction of the sample by the calibrator. Ratios < 0.8 were considered negative and ≥ 0.8 were considered positive.^21^ (4) IgM was measured using the automated analyser Vidas® 3 from BioMeriux Diagnostics (Cat. No. 423833, bioMérieux, France). The results interpretation of Vidas IgM according to test index value (i): *i* < 1.00 Negative (IgM antibodies to SARS-CoV-2 not detected), *i* ≥ 1.00 Positive (IgM antibodies to SARS-CoV-2 detected). Architect automated chemiluminescent assay (Abbott Laboratories, USA) was used to test the samples for the previous infection by measuring the SARS-CoV-2 anti-nucleoprotein IgG antibodies (anti-N), considering that the IgG antibodies produced against the RBD on the spike protein are different from the IgG antibodies produced against the nucleoprotein of the virus. Therefore, the positive anti-N results of SARS-CoV-2 anti-nucleoprotein IgG antibodies indicate previous exposure to the whole virus.^22^ Samples with previous infections were excluded from the VN group in this study.

### Statistical analysis

GraphPad Prism software (version 9.3.1, GraphPad Software, Inc., San Diego, CA, USA) was used to perform the statistical analysis. Continuous variables were summarized using geometric means and 95% confidence intervals (95% CIs). The collected dataset was subjected to the Shapiro–Wilk normality test to evaluate the data’s normality. Because of the absence of normal distribution, nonparametric tests were performed using Wilcoxon rank-sum test for pairwise group comparisons and Mann–Whitney U to test for the differences between independent samples. In the different scatter plots, the central horizontal bar line shows the geometric mean titre, and the error bars show the 95% CIs. All *P*-values were two-sided at a significance level of 0.05.

## Results

### Participants’ characteristics

A total of 140 subjects were included in this study, including 100 VN and 40 NI subjects. VN subjects (*n* = 100) had no previous history of infection (also anti-N negative) and received two doses of either BNT16b2 or mRNA-1273. After receiving the second dose in P-I and P-II, the median weeks were 6 (1.4 months) and 23 (5.3 months) weeks, respectively (Table 1). The VN group consisted of 40% females and 60% males. NI subjects (*n* = 40) were unvaccinated COVID-19-recovered individuals. In P-I 1 and P-II, the median weeks were 7 (~ 1.7 months) and 22 (5.2 months) weeks post-COVID-19 infection, respectively. The NI group comprised 20% females and 80% males (Table 1). Amongst the 40 NI subjects, 45% were symptomatic (18/40), 20% were paucisymptomatic (8/40), 20% were asymptomatic (8/40) and 15% had unspecified COVID-19 severity (6/40) (Table 1).

**Table 1:** Demographic characteristics of the study population

Characteristics	Vaccinated naïve (*n* = 100)		Naturally infected unvaccinated (*n* = 40)	
	P-I	P-II	P-I	P-II
Gender, *n* (%)				
Male	60/100 (60%)		32/40 (80%)	
Female	40/100 (40%)		8/40 (20%)	
Median age, years (IQR)	40 (30–47)		37 (30–40)	
Nationality^a^, *n* (%)				
Bangladeshi	0/100 (0%)		2/40 (5%)	
Egyptian	4/100 (4%)		12/40 (30%)	
Filipino	1/100 (1%)		3/40 (7.5%)	
Indian	8/100 (8%)		6/40 (15%)	
Jordanian	13/100 (13%)		4/40 (10%)	
Lebanese	5/100 (5%)		1/40 (2.5%)	
Nepalese	0/100 (0%)		2/40 (5%)	
Pakistani	4/100 (4%)		1/40 (2.5%)	
Qatari	11/100 (11%)		4/40 (10%)	
Sri Lankan	1/100 (1%)		1/40 (2.5%)	
Sudanese	2/100 (2%)		1/40 (2.5%)	
Tunisian	3/100 (3%)		2/40 (5%)	
Vietnamese	0/100 (0%)		1/40 (2.5%)	
Others^b^	48/100 (48%)		0%	
Median time of follow-up post-full vaccination, weeks (IQR)	5.7 (2.9–10.3)	22.6 (19.9–25.1)	NA	NA
Median time of follow-up post-full vaccination, months (IQR)	1.3 (1.3–2.6)	5.7 (5.0–6.3)	NA	NA
Median time of follow-up post-COVID-19 infection, weeks (IQR)	NA	NA	7.1 (5.6–8.6)	22.4 (20.2–24.3)
Median time of follow-up post-COVID-19 infection, months (IQR)	NA	NA	1.65 (1.3–2.0)	5.22 (4.7–5.7)
COVID-19 severity, *n* (%)				
Symptomatic	NA	NA	18/40 (45%)	
Pauci-symptomatic	NA	NA	8/40 (20%)	
Asymptomatic	NA	NA	8/40 (20%)	
Unspecified	NA	NA	6/40 (15%)	
Antibody positivity rate *n* (%)				
NTAb (IU/mL)	100/100 (100%)	99/100 (99%)	35/39 (90%)	35/39 (90%)
Anti S-RBD IgG (BAU/mL)	100/100 (100%)	100/100 (100%)	31/31 (100%)	30/31 (97%)
Anti S-RBD IgM ratio	12/47 (26%)	1/47 (2%)	17/40 (43%)	5/39 (13%)
Anti-S1 IgA ratio	92/93 (99%)	86/93 (92%)	23/32 (72%)	21/32 (66%)
Geometric mean antibody of levels (95% CI)				
NTAb (IU/mL)	1328.4 (1086.5–1624.2)	317.9 (261.5–386.5)	157.3 (108.7–227.7)	107.0 (80.4–142.3)
Anti S-RBD IgG (BAU/mL)	1950.9 (1617.5–2353.1)	366.1 (294.4–455.2)	66.5 (36.8–120.3)	58.65 (37.1–92.8)
Anti S-RBD IgM ratio	0.6 (0.4–0.8)	0.1 (0.0–0.3)	1.0 (0.6–1.5)	0.3 (0.3–0.4)
Anti-S1 IgA ratio	6.3 (5.4–7.3)	3.2 (2.6–3.9)	2.4 (1.5–3.7)	1.8 (1.2–2.7)

^a^Nationalities were chosen to represent the most populous groups in Qatar.

^b^Other nationalities in Qatar

NA; not applicable

### Antibody immune response assessment following vaccination

Amongst the VN participants in P-1, the positivity rates for anti-S-RBD IgG and NTAb antibodies were 100%. For anti-S1-IgA and anti-SRB-IgM, the positivity rates were 99 (92/93) and 26% (12/47), respectively. In P-II, 100% remained positive for anti-S-RBD IgG and 99% (99/100) were positive for NTAb antibodies. The anti-S1-IgA positivity rate dropped to 92% (86/93), and only 2% (1/47) remained positive for the IgM.

Amongst the NI participants in P-1, the positivity rate for NTAb antibodies was 90% (35/39). For anti-S-RBD-IgG, IgM and IgA, the positivity rates were 100 (31/31), 43 (17/40) and 72% (23/32), respectively. In P-II, 90% (35/39) were positive for NTAb antibodies. However, the positivity rates dropped to 97 (30/31), 13 (5/39) and 66 (21/32) for anti-S-RBD-IgG, -IgM and -IgA, respectively.

**Figure 1:**
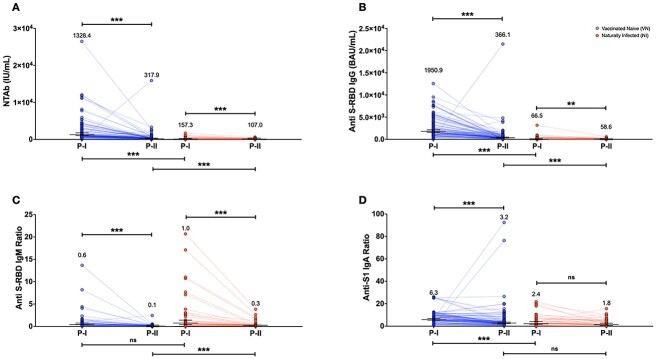
Antibody response measured parameters in P-1 and PII 2 in VN and NI groups. (A) NTAb total antibody levels measured by CL-900i® (IU/mL). (B) Anti-S-RBD IgG antibody levels (BAU/mL) measured by CL-900i®. (C) Anti-S-RBD-IgM measured by Vidas® 3. (D) Anti-S1 IgA ratios measured by Euroimmune. Plotted values above each group of points indicate the geometric mean. Black long and short horizontal bars indicate the geometric mean and 95% CI. Blue and red lines connect longitudinal samples from the same individual for VN and NI subjects, respectively. Statistical significance was determined using a two-tailed Wilcoxon matched-pairs signed-rank test. ^*^P ≤ 0.05, ^*^^*^P ≤ 0.01, ^*^^*^^*^P ≤ 0.001.

Amongst the VN group, waning in antibody levels was significantly observed in all measured parameters (Figure 1). A significant ~ 4-fold decrease (*P* < 0.001) in the levels of NTAb antibodies was observed, from the geometric mean 1328.4 (95% CI: 1086.5–1624.2) to 317.94 IU/mL (95% CI: 261.5–386.5 IU/mL) (Figure 1A). A significant ~ 5-fold reduction (*P* < 0.001) was observed in the levels of anti-S-RBD-IgG antibody levels from the geometric mean 1950.93 (95% CI: 1617.5–2353.1) to 366.107 BAU/mL (95% CI: 294.4–455.20) (Figure 1B). IgM declined by ~ 6-fold (*P* < 0.001) from the geometric mean 0.56 (95% CI: 0.4–0.8) to 0.09 (95% CI: 0.04–0.25) (Figure 1C). The levels of anti-S1-IgA antibodies decreased by ~ 2-fold (*P* < 0.0001) from the geometric mean 6.30 (95% CI: 5.4–7.3) to 3.19 (95% CI: 2.6–3.9) (Figure 1D).

Amongst the NI group, a waning in antibody levels was significantly observed in all measured parameters (Figure 1). A small but significant decrease in the levels of NTAb was observed (<2-fold *P* < 0.001), from the geometric mean 157.3 IU/mL, 95% CI: 108.7–227.7 to 107.0 IU/mL, 95% CI: 80.42–142.3 (Figure 1A). In addition, a small but significant decrease was also observed in the levels of anti-S-RBD-IgG antibody levels (<2-fold *P* < 0.001) from the geometric mean 66.54 (95% CI: 36.81–120.3) to 58.65 BAU/mL (95% CI: 37.06–92.80) (*p* = 0.005) (Figure 1B). IgM declined by ~ 3-fold from geometric mean 0.96 (95% CI: 0.6135–1.512) to 0.33 AU/mL (95% CI: 0.2527–0.4431) (*P* < 0.001) (Figure 1C). No significant decline was observed in anti-S1-IgA (Figure 1D).

## Discussion

To the best of our knowledge, this is the first study to comprehensively evaluate the levels of SARS-CoV-2 neutralizing, anti-S-RBD-IgG, Anti-S-RBD-IgM and anti-S1-IgA antibodies in VN and unvaccinated NI individuals. In the current study, mRNA vaccination elicited significantly greater NTAb, anti-S-RBD- IgG and anti-S1-IgA, compared with natural immunity (Figure 1). These results are in concordance with previous research findings reporting that mRNA vaccines induce higher antibody levels and greater antibody breadth than natural exposure to infection, and differences were particularly notable against the RBD domain.^23^ In addition, equally remarkable clinical trial data showed rapid induction of mRNA protective efficacy on a timescale similar to our current study.^3^^,^^5^

However, despite the mRNA vaccination-boosted antibody levels, this ‘boost’ was relatively short-lived, with ~ 2- to 6-fold significant waning observed in NTAb, anti-S-RBD-IgG, anti-S-RBD-IgM and anti-S1-IgA, 23 weeks post-full-vaccination (Figure 1). These results are in concordance with previous reports of a decline in humoral immune response 3–6 months post-full-vaccination.^24–26^ IgG and NTAb were reported to significantly decline 3 months post-vaccination by 7- and 4-fold, respectively.^24^^,^^25^ BNT162b2 and mRNA-1273 vaccines were reported to elicit potent IgA NTAb in serum after receiving two doses.^27^^,^^28^ However, consistent with our findings, it was reported that IgA declined over time.^29^

In comparison with mRNA vaccine-induced immunity, natural infection elicited a significant but less steady ~ 1–2-fold decline in NTAb, anti-S-RBD-IgG, and anti-S-RBD-IgM (Figure 1). Interestingly, however, unlike vaccination-induced immunity, a lasting IgA response was observed amongst NI individuals, with no significant decline observed after ~ 22 weeks post-SARS-CoV-2 infection (Figure 1). In addition, whereas anti-S1-IgA were ~ 3-fold higher in VN subjects compared with NI in P-I (<.001), anti-S1-IgA dropped to almost the same between the two groups, with no significant difference observed by the end of the follow-up period.

These findings are similar to a recent report showing that natural infection exhibit a lasting IgA response.^30^ Furthermore, anti-S1-IgA is known to dominate the early NTAb response in natural infection.^31^ Most importantly, it is believed that IgA antibodies can protect unvaccinated subjects against COVID-19.^32^ Recently, a study, which examined the patterns of humoral and cellular responses to SARS-CoV-2 for 6 months during the COVID-19 pandemic, reported that subjects who had IgA antibodies only never succumbed to COVID-19, as opposed to subjects with both IgG antibodies and T cells who contracted the disease.^32^ It is worth noting that IgA antibody responses in nasal secretions of volunteers infected with the common cold coronavirus 229E have been linked to shorter durations of viral shedding.^33^ Serum and salivary IgA antibody responses to SARS-Cov-2 spike antigens were recently reported,^34^ with salivary IgA antibodies lasting at least three months. Therefore, assessing the immune response of circulating anti-S1-IgA antibodies after vaccination is as important as testing for IgG and IgM.

This study had several limitations. It is known that recent variants, such as Omicron, need many folds of antibody titre to be neutralized compared to the original strains. That is, the mutations that emerged in different variants of the SARS-CoV-2 genome should have a significant influence on viral protein structures, shape, function and immunogenicity, which, in theory, should greatly affect the strength and the effectiveness of the immunological response and possibly duration of antibody’s waning in infected patients. It would be great to study the antibody response and waning against different variants. However, it was not applicable to include variant-specific data because of the limited number of samples and lack of sequencing data. In addition, a variety of variables could influence the level of immune response elicited after infection. It should be noted that our NI group included only 45% symptomatic subjects, whereas the remaining were paucisymptomatic (20%), asymptomatic (20%) or with unspecified severity (15%), which could have affected our results. In those with more severe COVID-19, NTAb antibody titers were reported to rise faster and reach a greater peak.^35–37^ Individuals with symptomatic SARS-CoV-2 infection have greater antibody titers than asymptomatic, and hospitalized people have higher antibody titers than those who are managed as outpatients.^35^^,^^36^^,^^38^^,^^39^

Furthermore, several studies have shown a link between cycle threshold (Ct) and antibody titre, with lower Ct values linked with greater antibody titers at the population level.^35^^,^^40^ These factors could have impacted the elicited immune response. Furthermore, we have not measured antibody levels prior to vaccine administration.

Despite these limitations, this study has several strengths that merit attention. First, most of the published studies have mainly focused on NTAb, IgG or IgM, whereas studies on IgA response are minimal, particularly amongst unvaccinated NI subjects. Second, in this study, we assessed anti-N antibodies, which is crucial to identify those who were exposed to a virus but were asymptomatic prior to vaccination, especially amongst those vaccinated with vaccines containing only S protein. In addition, despite the relatively small sample size across the analysed groups, we utilized strict inclusion criteria and included participants from a wide age range to achieve valid comparisons.

## Conclusion

Our findings provide important insights into the durability of vaccine- and natural infection-induced immunity. We evaluated the antibody responses of NTAb, anti-SRBD IgM, anti-S1-IgA and anti-SRBD IgG antibodies. Whereas double-dose mRNA vaccination elicited higher antibody titers compared with natural infection, this ‘boost’ was relatively short-lived in vaccinated individuals. In contrast, natural infection exhibited a less steady decline in NTAb antibodies, IgG, IgM and a lasting IgA response. Understanding the degree of waning immunity is crucial for policymaking, particularly regarding vaccination strategies, supporting the consideration of booster doses to sustain protection against COVID-19.

## Author Contributions

Conceptualization, H.A.S., H.M.Y., L.J.A.-R. and G.K.N.; methodology, H.A.S., B.A.H., S.Y. and F.M.S., software, B.A.H., S.Y. and F.M.S; validation, H.A.S., B.A.H., S.Y. and F.M.S.; formal analysis, H.A.S., G.K.N., B.A.H. and S.Y.; Investigation, H.A.S., A.I., H.M.Y., L.J.A.-R. and G.K.N.; resources, H.A.S., N.L., H.Q., N.A.D., H.M.Y., L.J.A.-R and G.K.N.; data curation, H.A.S., B.A.H., S.Y. and G.K.N.; writing—original draft preparation, H.A.S., S.Y. and G.K.N.; writing—review and editing, H.A.S., S.Y., N.Y., D.W.A., N.A.D., H.M.Y., L.J.A.-R and G.K.N; visualization, H.A.S., B.A.H., S.Y., N.Y., D.W.A., H.M.Y., L.J.A.-R A.I. and G.K.N; supervision, H.A.-S., H.M.Y. and G.K.N.; project administration, H.A.S., H.M.Y., L.J.A.-R and G.K.N.; funding, G.K.N. All authors have read and agreed to the published version of the manuscript.

## Data Availability

All data produced in the present study are available upon reasonable request to the authors.
